# 53BP1 Mediates the Fusion of Mammalian Telomeres Rendered Dysfunctional by DNA-PKcs Loss or Inhibition

**DOI:** 10.1371/journal.pone.0108731

**Published:** 2014-09-29

**Authors:** Ivana Rybanska-Spaeder, Rajib Ghosh, Sonia Franco

**Affiliations:** Department of Radiation Oncology and Molecular Radiation Sciences; and Department of Oncology; and the Sidney Kimmel Comprehensive Cancer Center, Johns Hopkins University School of Medicine, Baltimore, Maryland, United States of America; INSERM UMR S_910, France

## Abstract

Telomere dysfunction promotes genomic instability and carcinogenesis via inappropriate end-to-end chromosomal rearrangements, or telomere fusions. Previous work indicates that the DNA Damage Response (DDR) factor 53BP1 promotes the fusion of telomeres rendered dysfunctional by loss of TRF2, but is dispensable for the fusion of telomeres lacking Pot1 or critically shortened (in telomerase-deficient mice). Here, we examine a role for 53BP1 at telomeres rendered dysfunctional by loss or catalytic inhibition of DNA-PKcs. Using mouse embryonic fibroblasts lacking 53BP1 and/or DNA-PKcs, we show that 53BP1 deficiency suppresses G1-generated telomere fusions that normally accumulate in DNA-PKcs-deficient fibroblasts with passage. Likewise, we find that 53BP1 promotes telomere fusions during the replicative phases of the cell cycle in cells treated with the specific DNA-PKcs inhibitor NU7026. However, telomere fusions are not fully abrogated in DNA-PKcs-inhibited 53BP1-deficient cells, but occur with a frequency approximately 10-fold lower than in control 53BP1-proficient cells. Treatment with PARP inhibitors or PARP1 depletion abrogates residual fusions, while Ligase IV depletion has no measurable effect, suggesting that PARP1-dependent alternative end-joining operates at low efficiency at 53BP1-deficient, DNA-PKcs-inhibited telomeres. Finally, we have also examined the requirement for DDR factors ATM, MDC1 or H2AX in this context. We find that ATM loss or inhibition has no measurable effect on the frequency of NU7026-induced fusions in wild-type MEFs. Moreover, analysis of MEFs lacking both ATM and 53BP1 indicates that ATM is also dispensable for telomere fusions via PARP-dependent end-joining. In contrast, loss of either MDC1 or H2AX abrogates telomere fusions in response to DNA-PKcs inhibition, suggesting that these factors operate upstream of both 53BP1-dependent and -independent telomere rejoining. Together, these experiments define a novel requirement for 53BP1 in the fusions of DNA-PKcs-deficient telomeres throughout the cell cycle and uncover a Ligase IV-independent, PARP1-dependent pathway that fuses telomeres at reduced efficiency in the absence of 53BP1.

## Introduction

Mammalian chromosome ends are maintained by a nucleoprotein complex of TTAGGG repeats and the shelterin proteins (i.e., TRF1, TRF2, RAP1, TIN2, TPP1 and POT1) [1]. Loss of chromosome end capping due to critical telomere shortening or loss of shelterin function exposes telomeric DNA and activates the DNA Damage Response (DDR) [2]. DDR factors accumulate at telomere dysfunction-induced foci (TIFs) [3], where they signal cellular apoptosis or senescence, a protective response that prevents the propagation of cells with uncapped telomeres [4].

This protective response can however be thwarted by recruitment of end-joining factors that aberrantly “repair” dysfunctional telomeres by fusing them to other dysfunctional telomeres or to DSBs elsewhere [5]. Telomere fusions are thought to be highly deleterious, accelerating tissue and organismal ageing and promoting oncogenesis [6]. In the later context, telomere fusions amplify genomic instability by promoting the formation of complex chromosomal rearrangements via breakage-fusion-bridge (BFB) cycles [7]. In addition, telomere fusions promote aneuploidy via abnormal chromosome disjunction of fused chromosomes during mitosis, resulting in chromosomal gains [8].

The pathways that mediate the detection, signaling and fusion of dysfunctional telomeres are dictated by the mechanism of telomere dysfunction (i.e., the type of DNA lesion) and the stage of the cell cycle [1], [2]. In this context, TRF2-depleted telomeres in pre-replicative phases of the cell cycle are signaled via the ATM kinase and fused via canonical, ligase IV-dependent nonhomologous end-joining (C-NHEJ) [9], [10]. Similarly, catalytic inhibition of DNA-PKcs, a ubiquitous repair factor required for normal telomere maintenance [11]–[15], leads to ligase IV-dependent NHEJ of dysfunctional telomeres in the S/G2 phase of the cell cycle [16], suggesting that telomeres lacking DNA-PKcs may resemble a single-ended DSB. In contrast, dysfunctional telomeres in the context of POT1 loss evoke ATR-mediated signaling and are fused via “alternative” NHEJ (A-NHEJ) [9], a ligase IV-independent-pathway that rejoins DNA ends in an error-prone manner, sometimes using microhomologies [17]. Although the components of A-NHEJ pathway at telomeres are not fully elucidated, the fusion of shelterin-depleted telomeres in the absence of C-NHEJ relies on PARP1 and Ligase III [18], the same factors proposed to mediate A-NHEJ-mediated rearrangements of chromosomal DSBs elsewhere [19]–[21].

The choice between C-NHEJ and A-NHEJ-mediated repair is regulated in part via 53BP1, a BRCT and Tudor domain-containing protein that relocalizes to chromatin surrounding DSB [22] and to uncapped telomeres [3], [23]. Mechanistically, 53BP1 may facilitate C-NHEJ-mediated telomere fusions by promoting the spatial approximation of dysfunctional telomeres in far-apart chromosomes [23] and by suppressing DNA end resection [18], [24]. In support of this notion, ligase IV-dependent telomere fusions in TRF2-depleted cells are also dependent on 53BP1 [9], [23]. In contrast, ligase IV-independent telomere fusions in telomeres depleted of Pot1 or critically shortened occur efficiently in the absence of 53BP1 [9].

Here, we have taken a genetic approach to investigate a role for 53BP1 in the genesis of telomere fusions arising in cells lacking DNA-PKcs or treated with a DNA-PKcs catalytic inhibitor. While our work clearly demonstrates a role for 53BP1 throughout the cell cycle in this setting, it also uncovers an alternative PARP-dependent end-joining pathway that mediates fusions at lower efficiency in a 53BP1-independent manner.

## Materials and Methods

### Ethics statement

All experiments involving mice and generation of murine embryonic fibroblast (MEF) cell lines followed guidelines from the US Public Health *Policy on Humane Care and Use of Laboratory Animals*. The protocol for all experiments involving mice and the generation of MEF cell lines was approved by the Johns Hopkins University (JHU) Institutional Animal Care and Use Committee (IACUC; protocol permit number: MO11M270). All efforts were made to minimize animal suffering. This study did not involve work with human cells.

### Mice

Mice deficient for either 53BP1 (*Trp53bp1* ^−/−^; [25]), DNA-PKcs (*Prkdc* ^−/−^) [26], ATM (*Atm* ^−/−^; [27]); H2AX (*H2afx* ^−/−^ [28]) or PARP1 (*Parp1* ^−/−^ [29]) were previously described and kindly provided by Drs. Fred Alt, Junjie Chen and Ted Dawson. All mice had been backcrossed into a 129/Sv background. *Trp53bp1* ^−/−^ and *Prkdc* ^−/−^ mice were bred to generate *Trp53bp1* ^+/−^/*Prkdc* ^+/−^ mice and these were intercrossed to generate *Trp53bp1* ^−/−^/*Prkdc* ^−/−^ and corresponding controls. All experiments followed guidelines from the US Public Health *Policy on Humane Care and Use of Laboratory Animals*. The protocol for all experiments in this specific study was approved by the Johns Hopkins University (JHU) Institutional Animal Care and Use Committee (IACUC; protocol permit number: MO11M270). All efforts were made to minimize suffering.

### Cell culture

We obtained E13.5 mouse embryonic fibroblasts (MEFs) from timed matings, following standard protocols. All MEF lines were derived in compliance with guidelines from the Johns Hopkins University (JHU) Institutional Animal Care and Use Committee (IACUC; protocol permit number: MO11M270). MEFs lacking MDC1 (*Mdc1* ^−/−^) were a kind gift of Dr. Zhenkun Lou (Mayo Clinic, Rochester MN) [30]. MEFs were grown in 10% FCS/DMEM supplemented with sodium pyruvate, L-glutamine and penicillin/streptomycin. MEFs were passaged according to a standard 3T3 protocol until immortalized.

### Treatments with DNA repair inhibitors

Exponentially growing MEF cultures were treated with 40 µM NU7026 (Calbiochem, 260961) and metaphases obtained after 24 hours. Colcemid (10 µg/mL) was added during the last 4 hours of the treatment period. For PARP inhibition, cells were co-incubated with either 1 µM olaparib (Selleckchem, S1060) or 3 µM veliparib/ABT-888 (Enzo Life Sciences, ALX-270-444-M001). For ATM inhibition, we used 10 µM KU55933 (InSolution ATM kinase Inhibitor, Calbiochem, #118502).

### Generation of shRNAs and lentiviral infection

To deplete Ligase IV, we obtained five shRNAs from Sigma (TRCN0000071158, TRCN0000071159, TRCN0000071160 TRCN0000071161 and TRCN0000071162). As detailed in the manuscript, TRCN0000071159 and TRCN0000071162 were found to induce the most efficient “knock down” and were used for experiments. As a control, we used an shRNA to the Green Fluorescent Protein (GFP; Sigma). To deplete PARP1, we cloned a previously described shRNA (targeting the sequence GGCCCTTGGAAACATGTATG; [18]) into pLKO.1 using standard procedures. Briefly, the forward oligonucleotide (5′ ccggGGCCCTTGGAAACATGTATGctcgagCATACATGTTTCCAAGGGCCtttttg—3′) and the reverse oligonucleotide (5 aattcaaaaaGGCCCTTGGAAACATGTATGctcgagCATACATGTTTCCAAGGGCC‘3′) were co-denatured, reannealed and cloned into AgeI/EcoRI-digested pLKO.1 vector. Clones containing restriction fragments of the appropriate sizes were verified by sequencing with the pLKO.1 primer 5′ CAAGGCTGTAGAGATAATTGGA 3′. For lentiviral infection of MEFs, shRNAs-expressing vectors and packaging plasmids were co-transfected into HEK293T cells using Fugene 6 Transfection Reagent (Promega). MEFs were incubated with filtered supernatants overnight for two cycles and selected in puromycin (2 µg/mL) for three days prior to analysis.

### Proliferation assay

After lentiviral infection, MEFs were seeded in 6-well plates in triplicates (7.5×10^4^ cells/well). After allowing for attachment overnight, cells were either mock-irradiated or irradiated using a CIXD X-Ray irradiator (dual x-ray tube system), Xstrahl Ltd., UK operating at a dose rate of 3.93 Gy/min. After irradiation, cells were trypsinized and counted at the indicated timepoints.

### Telomere FISH analysis

To prepare metaphases, MEFs were incubated in 0.1 µg/mL colcemid (KaryoMAX, Gibco) for 4 hr, swollen in 30 mM sodium citrate for 25 min at 37°C and fixed in methanol/acetic acid (3/1). Metaphases were hybridized with a telomere probe as described [31]. Briefly, slides were fixed in 4% formaldehyde and digested in pepsin prior to denaturation at 80°C for 3 min. Slides were then hybridized with a Cy3-labeled telomeric (TTAGGG)_3_ probe (Applied Biosystems), washed and mounted in Vectashield with DAPI (Vector Laboratories, Burlingame, California). Images were obtained using a Zeiss Axioplan Imager Z.1 microscope equipped with a Zeiss AxioCam and an HXP120 mercury lamp (Jena GmbH) and dedicated software (Zeiss Axiovision Rel 4.6). Because the number of fusions varies significantly from cell to cell in a typical experiment, data is presented in histograms as the distribution of number of telomere fusions per cell throughout the manuscript.

### Real Time Reverse Transcription Polymerase Chain Reaction (RT-PCR)

MEFs were resuspended in Trizol and RNA extracted following manufacturer's protocols. Two µg of RNA were reverse transcribed using RT-III (Invitrogen) and cDNA was amplified using Power Sybr Green PCR Master Mix in a 7900HT Fast Real-Time PCR System with SDS v2.3 software. Data was analyzed using RQ Manager v1.2, all from Applied Biosystems (Carlsbad, California). Primers were: Lig4-F: 5′-CCGGGTGAAGAAATCGTGTC-3′; Lig4-R: 5′-CCTTTCTGTATCCGTTCTAGTGTG-3′; Gapdh-F: 5′-CATGGCCTTCCGTGTTCCTA-3′; Gapdh-R: 5′- TGCCTGCTTCACCACCTTCT-3′.

### Immunoblotting

Cells were resuspended in RIPA buffer and protein transferred to PVDF membranes as described [8]. PARP1 was detected using a mouse monoclonal antibody (clone C-2-10; Novus Biologicals, NB100-112; 1∶5000). To control for loading, membranes were probed with a mouse monoclonal antibody to α-tubulin (Millipore; 1∶5000). Membranes were incubated in Amersham ECL Prime Western Blotting Detection Reagent (RPN2232; GE Healthcare) and chemiluminescence quantified in a GelDoc apparatus using Quantity One software under Chemi Hi Sensitivity settings (all from BioRad, Hercules, CA).

### Statistical analyses

Statistical significance was calculated using Student's t test. At least 3 data points obtained from at least 3 independent experiments were used for each calculation.

## Results

### 53BP1 promotes telomere fusions in DNA-PKcs-deficient MEFs during the G1 phase of the cell cycle

**Figure 1:**
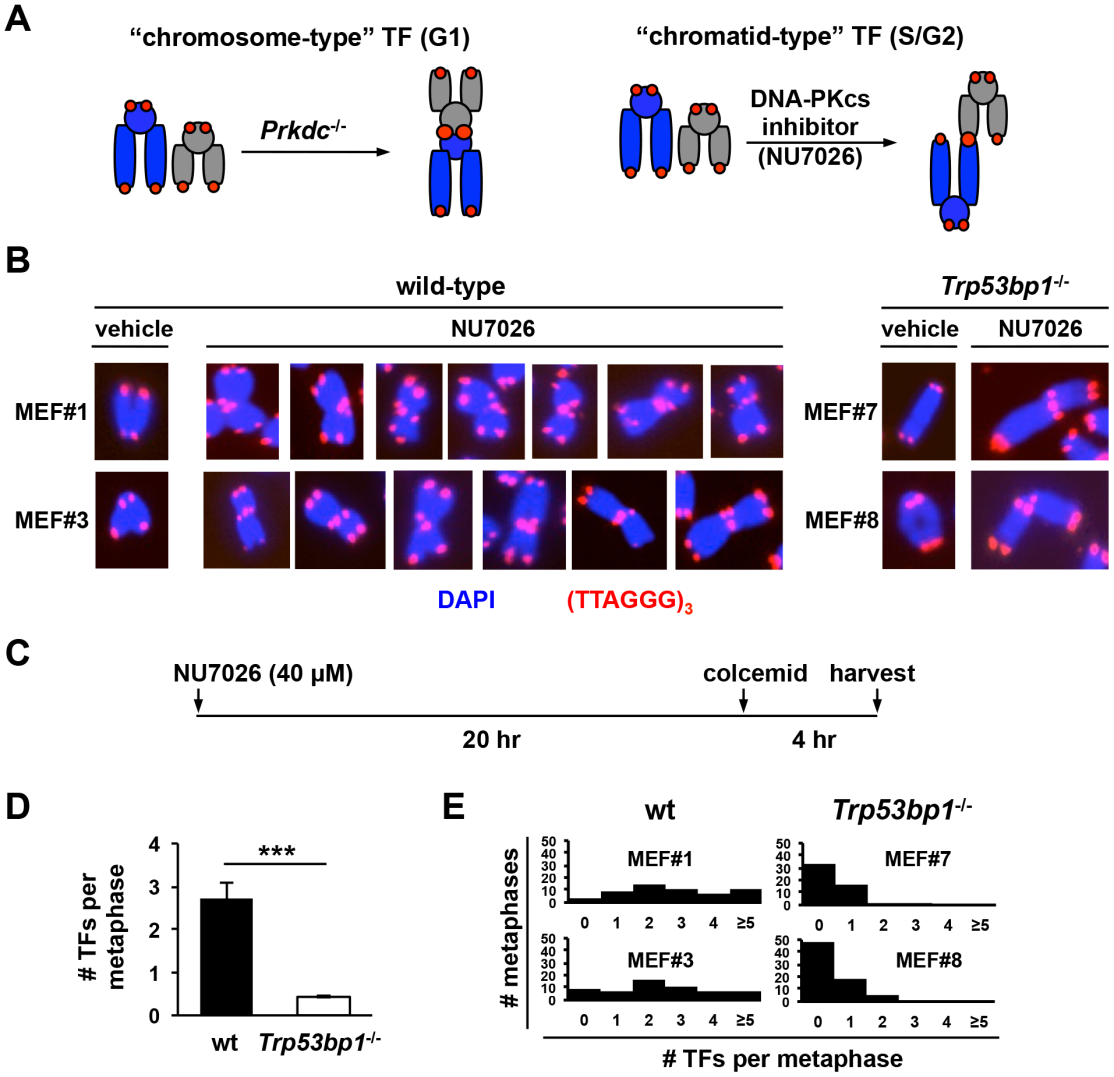
53BP1 promotes telomere fusions in cells treated with a DNA-PKcs inhibitor. (**A**) Schematic of types of telomere fusions observed in this study. Left panel: most fusion in DNA-PKcs null mouse cells (*Prkdc* ^−/−^) are “chromosome-type” (two telomere signals at the fusion point). Right panel: in contrast, most fusions in wt cells treated with the DNA-PKcs inhibitor NU7026 occur in replicative phases of the cell cycle and are therefore “chromatid-type” (three telomere signals). (**B**) Examples of “chromatid-type” telomere fusions in wt and 53BP1-deficient cells treated with the DNA-PKcs inhibitor NU7026 for 24 hours. Normal chromosomes in cells treated with vehicle (DMSO) are shown for comparison. (**C–E**) Quantification of NU7026-induced telomere fusions in wt and *Trp53bp1* ^−/−^ MEFs. Exponentially growing cells were incubated with 40 µM NU7026 or vehicle (DMSO) for 24 hours. Colcemid was added during the last four hours of the incubation period (diagrammed in C). Cells were fixed and metaphases analyzed by telomere FISH. The number of telomere fusions per metaphase in NU7026-treated cells is shown in D. Bars represent the average and standard deviation of two independent experiment (n = 2 MEF lines per experiment). The distribution of the number of telomere fusions per metaphase in the same experiments is shown in E.

Rare telomere fusions occur spontaneously in primary DNA-PKcs-deficient mouse embryonic fibroblasts (MEFs) or B lymphocytes (approximately 0.2 telomere fusions/metaphase) [12], [32]. In MEFs, the frequency of telomere fusions increases with passage, and immortalized DNA-PKcs-deficient MEFs often harbor multiple telomere fusions per cell [8], [11]–[15]. This accumulation of telomere fusions with passage is thought to result from the loss of a p53-dependent checkpoint that normally prevents fusions in primary cells [8], [11]–[15]. In support of this notion, we recently reported that p53 similarly suppresses telomere fusions in DNA-PKcs-deficient T cell lymphomas [8]. Regardless of cell lineage, spontaneous telomere fusions observed in DNA-PKcs-deficient cells typically involve nonhomologous chromosomes and are “chromosome-type” (involving the two chromatids; see Fig. 1A for diagram) [8], [11]–[15], indicating that they occur in G1 and are replicated upon cell cycle progression [33].

To investigate a requirement for 53BP1 in the fusion of telomeres lacking DNA-PKcs, we took a genetic approach and generated DNA-PKcs-deficient mice in 53BP1-proficient or deficient backgrounds, by breeding of single mutants (*Trp53bp1* ^−/−^/*Prkdc* ^−/−^ mice and cells). Consistent with mostly epistatic roles for 53BP1 and DNA-PKcs in C-NHEJ [1], [34], *Trp53bp1* ^−/−^/*Prkdc* ^−/−^ mice are viable, fertile and show no significant additional phenotype in growth and development over single mutants when followed for up to 6 months of age (not shown).

To quantify telomere fusions, we performed telomere FISH (T-FISH) on metaphase spreads of *Trp53bp1* ^−/−^/*Prkdc* ^−/−^ MEFs (n = 2 embryos) and control littermate *Trp53bp1* ^+/−^/*Prkdc* ^−/−^ (n = 2), *Trp53bp1* ^−/−^/*Prkdc* ^+/−^ (n = 3) and *Trp53bp1* ^+/−^/*Prkdc* ^+/−^ (n = 1) MEFs (Table 1). For all embryos, we analyzed both primary, early passage (P2–3) and passage-immortalized (P29–63) cultures. Consistent with previous reports [11]–[15], [23], [32], [35], loss of DNA-PKcs but not 53BP1 resulted in “chromosome-type” telomere fusions (Table 1). Telomere fusions in DNA-PKcs null MEFs were rare at early passage (one fusion in 84 metaphases; 0.01 fusion/metaphase) but abundant in immortalized cells (55 fusions in 58 metaphases; 0.7 fusions/cell on average), validating the use of our DNA-PKcs null strain for these studies. Significantly, analyses of *Trp53bp1* ^−/−^/*Prkdc* ^−/−^ MEFs generated and analyzed in parallel to single mutant controls revealed no telomere fusions in 73 early-passage or 120 passage-immortalized metaphases, even when examined at P63 (Table 1). We conclude that 53BP1 is required for the formation of spontaneous telomere fusions arising in DNA-PKcs-deficient cells.

**Table 1:** Analysis of telomere instability in MEFs deficient for DNA-PKcs and/or 53BP1 (*Prkdc*/*Trp53bp1* MEFs).

MEF ID	Genotype	Passage	# metaphases	# TFs		#TFs/metaphase	
				chromosome-type	chromatid-type	chromosome-type	chromatid-type
*Primary MEFs*							
8	*Prkdc* ^+/−^/*Trp53bp1* ^+/−^	P3	19	0	0	0	0
9	*Prkdc* ^+/−^/*Trp53bp1* ^−/−^	P3	29	0	0	0	0
1	*Prkdc* ^+/−^/*Trp53bp1* ^−/−^	P2	30	0	0	0	0
6	*Prkdc* ^−/−^/*Trp53bp1* ^+/−^	P3	54	1	0	0.02	0
5	*Prkdc* ^−/−^/*Trp53bp1* ^+/−^	P2	30	0	0	0	0
4	*Prkdc* ^−/−^/*Trp53bp1* ^−/−^	P3	43	0	0	0	0
3	*Prkdc* ^−/−^/*Trp53bp1* ^−/−^	P2	30	0	0	0	0
*Passage-immortalized MEFs*							
8	*Prkdc* ^+/−^/*Trp53bp1* ^+/−^	P43	29	1	0	0.03	0
8	*Prkdc* ^+/−^/*Trp53bp1* ^+/−^	P63	30	0	0	0	0
9	*Prkdc* ^+/−^/*Trp53bp1* ^−/−^	P43	28	0	0	0	0
9	*Prkdc* ^+/−^/*Trp53bp1* ^−/−^	P63	30	0	0	0	0
10-2	*Prkdc* ^+/−^ *Trp53bp1* ^−/−^	P60	20	0	0	0	0
1	*Prkdc* ^+/−^/*Trp53bp1* ^−/−^	P29	30	0	0	0	0
6	*Prkdc* ^−/−^/*Trp53bp1* ^+/−^	P43	26	7	0	0.26	0
6	*Prkdc* ^−/−^/*Trp53bp1* ^+/−^	P63	30	13	0	0.43	0
8-4	*Prkdc* ^−/−^/*Trp53bp1* ^+/−^	P60	22	35	0	1.59	0
4	*Prkdc* ^−/−^/*Trp53bp1* ^−/−^	P43	30	0	0	0	0
4	*Prkdc* ^−/−^/*Trp53bp1* ^−/−^	P63	30	0	0	0	0
3	*Prkdc* ^−/−^/*Trp53bp1* ^−/−^	P43	30	0	0	0	0
3	*Prkdc* ^−/−^/*Trp53bp1* ^−/−^	P63	30	0	0	0	0

Metaphases were stained with a telomere probe and scored for telomere fusions (TFs). TFs were further classified as “chromosome-type” or “chromatid-type”.

### 53BP1 suppresses telomere fusions that arise in DNA-PKcs-inhibited cells undergoing replication

The C-terminal PI3 kinase-like domain of DNA-PKcs mediates the phosphorylation of serine and threonine residues in DNA-PKcs itself as wells as other substrates [36], [37]. Small molecules that inhibit this catalytic domain with high specificity have been developed [38], including NU7026 [39] and IC86621 [40]. Using these inhibitors, previous work established that the catalytic function of DNA-PKcs is indeed required for its functions in the suppression of telomere fusions [41], [42]. Specifically, treatment of wt MEFs with either NU7026 or IC86621 rapidly induces telomere fusions [41], [42]. Moreover, while loss of DNA-PKcs induces “chromosome type” fusions in G1 (see above), DNA-PKcs inhibition induces replication-related “chromatid-type” fusions (see Fig. 1A for schematic; Fig. 1B and Fig. S1 for examples). These replication-associated fusions are nevertheless mediated via C-NHEJ to a great extent, because depletion of ligase IV markedly reduces their frequency [41]. In contrast, a role for 53BP1 in this setting has not been examined.

To determine whether 53BP1 is required for the fusion of DNA-PKcs inhibited telomeres, we next quantified telomere fusions in NU7026-treated, immortalized *Trp53bp1* ^−/−^ and control wt MEFs via T-FISH (n = 2 independent lines per genotype; Table 2, Fig. 1C for experimental design; Fig. 1D–E for quantification). As expected [41], NU7026 induced frequent chromatid fusions with a robust telomere signal at the fusion point in wt cells (315 fusion in 116 metaphases, or 2.7 fusion/metaphase; Table 2, Fig. 1D). In contrast, although telomere fusions were observed in *Trp53bp1* ^−/−^ MEFs, they were significantly less frequent (54 in 128 metaphases, or 0.4 fusions per metaphase; Table 2, Fig. 1D). This difference was highly significant (p<0.001; n = 4 independent experiments). Moreover, while wt MEFs containing fusions often harbored multiple (>3) fusions, *Trp53bp1* ^−/−^ MEFs harboring fusions typically showed only one or two fusions per cell (see histograms in Fig. 1E).

**Table 2:** Genetic requirements for the formation of NU7026-induced telomere fusions (TF) in mouse fibroblasts.

MEF ID	Genotype	Additional treatments*	Vehicle			NU7026 (40 uM)		
			# metaphases	# TFs	# TFs/metaphase	# metaphases	# TFs	#TFs/metaphase
#1	wt		28	0	0.0	57	169	2.96
#3	wt		27	0	0.0	59	146	2.47
#7	*Trp53bp1* ^−/−^		30	0	0.0	54	23	0.43
#8	*Trp53bp1* ^−/−^		30	0	0.0	74	31	0.42
#PB-3	*Trp53bp1* ^−/−^/*Prkdc^−/−^*		20	0	0.0	20	0	0.0
#PB-4	*Trp53bp1* ^−/−^/*Prkdc^−/−^*		20	0	0.0	20	0	0.0
#1	wt	sh-GFP	-	-	-	35	42	1.2
#1	wt	sh-Lig4#	-	-	-	60	17	0.28
#7	*Trp53bp1* ^−/−^	sh-GFP	-	-	-	28	10	0.36
#7	*Trp53bp1* ^−/−^	sh-Lig4#	-	-	-	43	8	0.18
#8	*Trp53bp1* ^−/−^	sh-GFP	-	-	-	30	12	0.4
#8	*Trp53bp1* ^−/−^	sh-Lig4#	-	-	-	59	16	0.27
#1	wt	vehicle	30	0	0.0	60	85	1.42
#7	*Trp53bp1* ^−/−^	vehicle	30	0	0.0	56	14	0.25
#8	*Trp53bp1* ^−/−^	vehicle	24	0	0.0	46	11	0.24
#1	wt	olaparib	30	0	0.0	60	85	1.42
#7	*Trp53bp1* ^−/−^	olaparib	30	0	0.0	55	0	0.0
#8	*Trp53bp1* ^−/−^	olaparib	30	0	0.0	47	0	0.0
#1	wt	veliparib	20	0	0.0	30	39	1.3
#7	*Trp53bp1* ^−/−^	veliparib	20	0	0.0	20	0	0.0
#8	*Trp53bp1* ^−/−^	veliparib	20	0	0.0	16	0	0.0
#1	wt		-	-	-	28	43	1.54
#P1–3	*Parp1^−/−^*		30	0	0.0	30	35	1.17
#P1–5	*Parp1^−/−^*		30	0	0.0	30	40	1.33
#2	*Parp1^−/−^/Prkdc^−/−^*		22	0	0.0	22	0	0.0
#1	wt		-	-	-	30	82	2.73
#ATM-3	*Atm* ^−/−^		20	0	0.0	30	67	2.23
#1	wt	vehicle	-	-	-	20	27	1.35
#1	wt	KU55933	30	0	0.0	26	31	1.19
#AB-4	*Atm* ^−/−^/*Trp53bp1* ^−/−^		20	0	0.0	30	11	0.37
#AB-4	*Atm* ^−/−^/*Trp53bp1* ^−/−^	olaparib	-	-	-	28	0	0.0
#AB-8	*Atm* ^−/−^/*Trp53bp1* ^−/−^		20	0	0.0	30	12	0.4
#AB-8	*Atm* ^−/−^/*Trp53bp1* ^−/−^	olaparib	-	-	-	31	0	0
#1	wt		-	-	-	30	55	1.83
#M-1	*Mdc1−/−*		25	0	0.0	25	0	0.0
#1	wt		30	0	0.0	20	23	1.15
#H-2	*H2ax−/−*		30	0	0.0	0	0.0	0
#H-16	*H2ax−/−*		30	0	0.0	0	0.0	0

Metaphases were stained with a Cy3-labeled (TTAGGG)_3_ probe prior to scoring for “chromatid-type” TFs.

*Additional treatments: PARP inhibitors: olaparib (1 µM); veliparib (3 µM). ATM inhibitor: KU55933(10 µM).

# Pooled data from two independent shRNAs (sh-Lig4 59 and sh-Lig4 62; see Fig. 3).

The residual fusions in the absence of 53BP1 were due to DNA-PKcs inhibition and not to an off-target effect, because NU7026 failed to induce “chromatid-type” telomere fusions in MEFs deficient for DNA-PKcs or doubly deficient for DNA-PKcs and 53BP1 (Table 2). Together with our observations in *Trp53bp1* ^−/−^/*Prkdc* ^−/−^ MEFs above, these findings indicate that 53BP1 promotes the fusions of DNA-PKcs-deficient telomeres throughout the cell cycle.

### 53BP1 and ligase IV function in an epistatic pathway in the fusion of DNA-PKcs-deficient telomeres

**Figure 2:**
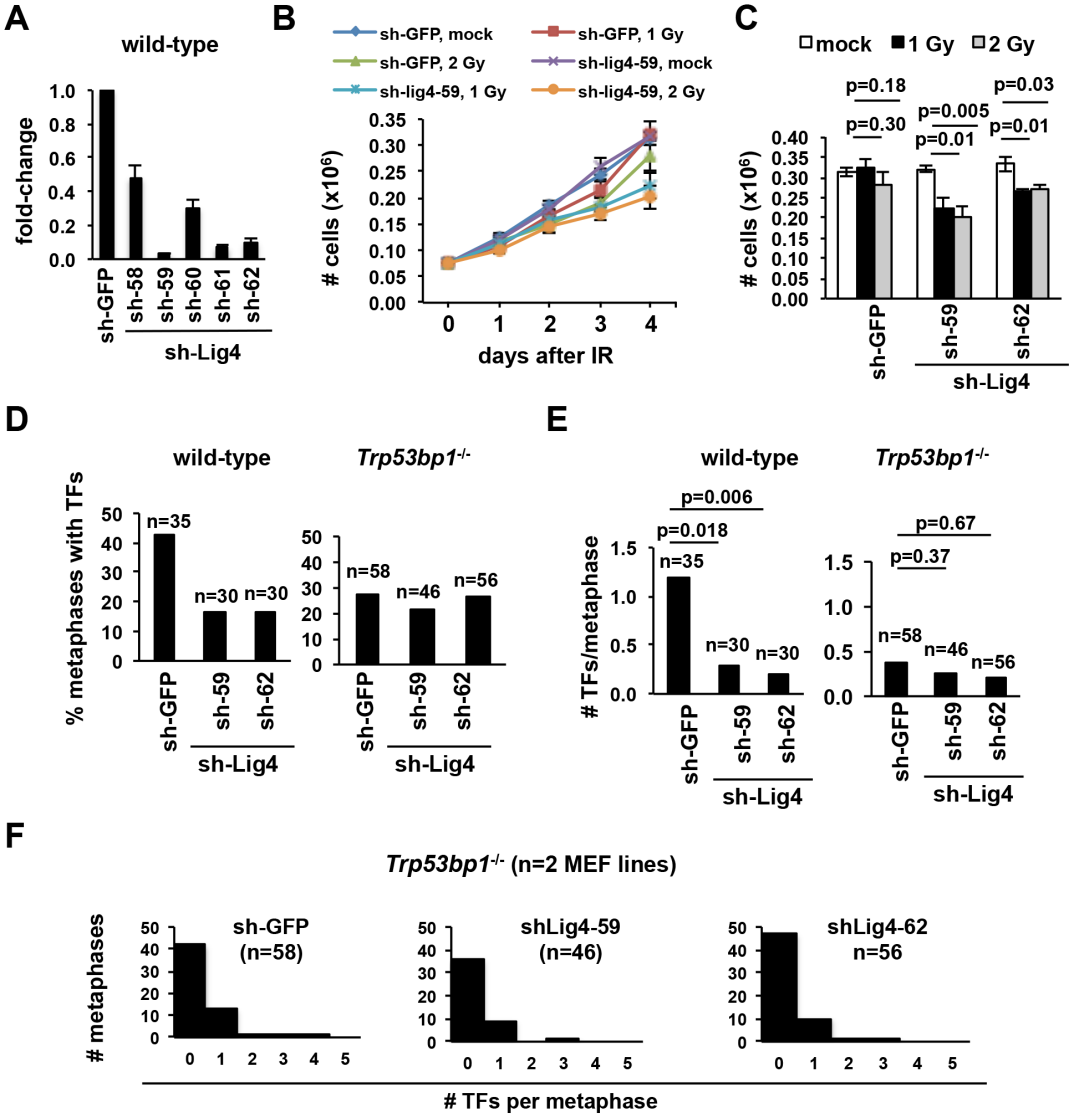
Ligase IV and 53BP1 function in an epistatic manner in the fusion of DNA-PKcs-deficient telomeres. (**A**) Wt MEFs were infected with either of five shRNAs to Lig4 or a control shRNA to GFP and, after selection, Lig4 RNA was quantified by Real Time RT-PCR. (**B–C**) After Lig4 “knock down” with the indicated shRNAs, cells were exposed to either 1 or 2 Gy of IR and counted daily, in triplicates. The total number of cells at 1, 2, 3 or 4 days after IR in sh-Lig4-59 cells is shown in B. Similar results were obtained with sh-Lig4-62 (not shown). The total numbers of cells at day 4 in cells expressing either shRNA are indicated in (C). Bars represent the average and standard deviation of triplicates. (**D–F**) After Ligase IV “knock down” with the indicated shRNAs, cells were treated with NU7026 for 24 hours and the number of telomere fusions quantified by telomere FISH. The percentage of metaphases containing telomere fusions is shown in D and the number of telomere fusions per metaphase is shown in E (bars; p values are indicated) and F (histograms). Data represents a pool of 2 independent MEF cultures.

53BP1 promotes ligase IV-dependent canonical NHEJ of distant breaks at antigen receptor loci [43] and at TRF2-depleted telomeres [23]. In the latter scenario, 53BP1 is thought to bring together far-apart telomeres by increasing chromatin mobility [23], a prerequisite for ligation. In contrast, telomere fusions mediated via ligase IV-independent pathways do not require 53BP1 [9]. Based on these previous observations, we next analyzed whether 53BP1 and ligase IV may similarly function in an epistatic manner at DNA-PKcs-inhibited telomeres (Fig. 2).

To deplete Ligase IV, we infected wt MEFs with lentiviral vectors expressing either of five shRNAs to Ligase IV and quantified the extent of “knock-down” relative to control cells infected with an shRNA to GFP (Fig. 2A). Because small amounts of residual ligase may be sufficient for C-NHEJ, we next validated two shRNAs that “knocked down” Ligase IV efficiently (sh-Lig4-59 and sh-Lig4-62) using functional assays (Fig. 2B–E). In this context, expression of either shRNA aggravated IR-induced growth arrest (Fig. 2B–C), consistent with defective DSB repair by C-NHEJ in the absence of Ligase IV. To validate the knock-down of Ligase IV at the telomere, we next quantified telomere fusions in NU7026-treated “knocked down” MEFs. Consistent with previous observations in *Lig4* ^−/−^ MEFs [41], the number of telomere fusions was markedly diminished upon ligase IV depletion (Fig. 2D–E). Specifically, the percentage of metaphases containing at least one telomere fusions was 42.9%, 16.7% and 16.7% for MEFs expressing shGFP, sh-Lig4-59 and sh-Lig4-62, respectively (Fig. 2D). Similarly, the number of telomere fusions per metaphase for the same cultures was 1.2, 0.3 and 0.2, respectively (Fig. 2E). These differences were statistically significant (see Fig. 2E for p values).

Using these reagents, we next quantified telomere fusions in 53BP1-deficient MEFs depleted of Ligase IV. The frequency of telomere fusions in these cultures was comparable to a control 53BP1-deficient culture expressing shGFP (Fig. 2D for percentage of metaphases containing telomere fusions; Fig. 2E for number of telomere fusions per cell and p values; Fig. 2F for histogram of pooled cultures). We conclude that 53BP1 and Ligase IV likely function in an epistatic pathway in the generation of telomere fusions at DNA-PKcs-inhibited telomeres.

### A PARP1-dependent end-joining pathway mediates 53BP1-independent telomere fusions in S/G2

While our analyses above establish a major role for 53BP1 in the generation of ligase IV-dependent fusions, we consistently observed residual telomere fusions in 53BP1-deficient cells, indicating that it is not essential. The frequency of 53BP1-independent telomere fusions is however approximately 10-fold lower than in 53BP1-proficient cells (Table 2, Fig. 1D), suggesting that they may be mediated via an alternative, low-efficiency end-joining pathway (A-NHEJ) [17].

**Figure 3:**
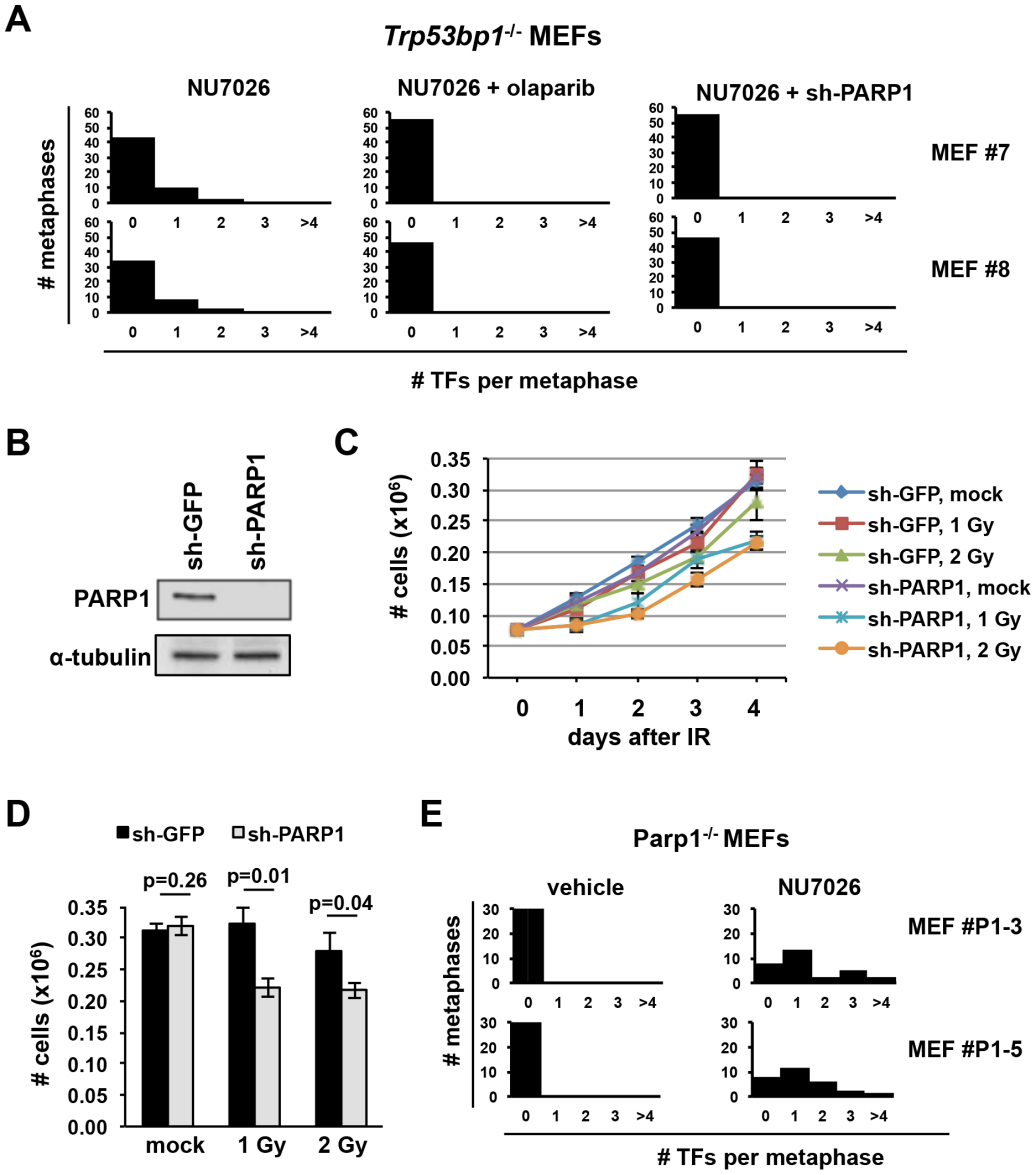
A PARP1-dependent pathway promotes telomere fusions in 53BP1-deficient but not in wt cells. (**A**) Two independent lines of 53BP1-deficient cells (MEF #7 and MEF #8) were treated with 40 µM NU7026 in the presence of either 1 µM olaparib (or vehicle) for 24 hours, or after “knocking down” PARP1 using a specific shRNA (sh-PARP1). Metaphases were hybridized with a telomere probe and telomere fusions were recorded. Histograms show the distribution of the number of telomere fusions per metaphase for each culture and treatment condition. Olaparib alone did not induce telomere fusions (not shown). (**B**) To validate the “knock down”, extracts of wt MEFs expressing either sh-PARP1 or a control sh-GFP were probed with an antibody to PARP1. α-tubulin was used as a loading control. (**C–D**) To functionally validate the “knock down”, cells were treated with either 1 or 2 Gy of IR and counted. Total cell number at days 1–4 after IR is shown in C. Total cell number at day 4; bars represent the average and standard deviation of triplicates. (**E**) Two independent lines of PARP1-deficient MEFs (MEF#P1–3 and MEF#P1–5) were treated with 40 µM NU7026 or vehicle (DMSO) for 24 hours and telomere fusions quantified in metaphase spreads. Histograms show the distribution of the number of telomere fusions per metaphase for each culture and treatment condition.

Poly(ADP)ribose polymerase 1 (PARP1) promotes (A-NHEJ) at DSBs [19], [21], [44], [45] and at shelterin-depleted telomeres [18]. To test whether PARP1 may mediate the fusion of telomeres in 53BP1-deficient, DNA-PKcs-inhibited cells, we quantified telomere fusions in cells exposed to 1 µM olaparib, a *bona fide* PARP inhibitor [46]. In line with our findings above, treatment of 53BP1-deficient cells with NU7026 plus vehicle resulted in a low frequency of telomere fusions (25 fusions in 102 metaphases, or 0.25 fusions/metaphase; Table 2; Fig. 3A). In contrast, we detected no fusions in cells incubated with both NU7026 and olaparib (n = 2 independent MEF lines; Table 2, Fig. 3A). These observations were not due to an unspecific effect of olaparib, because treatment with veliparib/ABT-888, a second well-validated PARP inhibitor [46], similarly abrogated NU7026-induced telomere fusions in 53BP1-deficient cells (n = 2 independent lines; Table 2). In contrast to its effect on 53BP1-deficient cells, olaparib or veliparib treatment had no measurable effect on the frequency of NU7026-induced fusions in wt cells (Table 2), consistent with previous observations that PARP activity is dispensable for C-NHEJ-mediated repair [47], [48].

The fusion of shelterin-depleted telomeres via an “alternative” pathway relies on PARP1 [18]. To determine whether residual fusions in NU7026-treated 53BP1-deficient cells require PARP1, we repeated these experiments in 53BP1-deficient cells “knocked down” for PARP1 or, as a control, GFP (Fig. 3A–D). The shRNA to PARP1 used in these experiments (sh-PARP1) was previously shown to abrogate telomere fusions in shelterin-depleted telomeres [18]. Expression of this shRNA in MEFs resulted in protein depletion by immunoblotting (Fig. 3B) and, as expected of a functional “knock down” [49], increased sensitivity to IR (Fig. 3C–D), consistent with a Significantly, we observed no telomere fusions in NU7026-treated *Trp53bp1* ^−/−^ MEFs expressing sh-PARP1 (n = 9 metaphases from 2 independent embryos). In contrast, the frequency and distribution of NU7026-induced telomere fusions was similar in wt and *Parp1* ^−/−^ MEFs (Table 2; Fig. 3E), indicating that PARP1 is dispensable for C-NHEJ-mediated telomere fusions when 53BP1 is present. The telomere fusions observed in NU7026-treated *Parp1* ^−/−^ cells were indeed mediated by DNA-PKcs-inhibition, because *Parp1* ^−/−^ cells treated with vehicle showed no telomere fusions (not shown) and NU7026 treatment failed to induce “chromatid-type” telomere fusions in MEFs lacking both PARP1 and DNA-PKcs (*Parp1* ^−/−^/*Prkdc* ^−/−^ MEFs; Table 2).

Altogether, these observations point to distinct genetic requirements for the generation of telomere fusions in 53BP1-proficient and -deficient backgrounds. Moreover, the observation that PARP inhibitor treatment or PARP1 “knock down” abrogate residual fusions in 53BP1-deficient cells suggest that PARP1 may be a key mediator in the alternative pathway at DNA-PKcs-inhibited telomeres.

### H2AX and MDC, but not ATM, are required for telomere fusions in DNA-PKcs-inhibited cells

Telomere fusions occur in the context of modified chromatin, generally referred to as Telomere dysfunction-Induced Foci (TIF) [3]. TIF factors mediate the detection and signaling of dysfunctional telomeres, coordinating the molecular events that eventually lead to their covalent attachment [1]. In this context, the ATM kinase functions in the detection and signaling of DSBs and dysfunctional telomeres [1], [50]. ATM was previously shown to modulate telomere fusions formation in a cell cycle regulated manner: it promotes the fusion of TRF2-depleted telomeres in G1, but mildly suppresses telomere fusions during replication [51], when ATR is the predominant regulatory kinase. ATM substrates at telomeres include histone H2AX, a constitutive component of the nucleosome [52]. H2AX is phosphorylated (to form γ-H2AX) in response to telomere dysfunction [3], a modification that promotes telomere fusions via recruitment of MDC1 [53] an other downstream factors.

**Figure 4:**
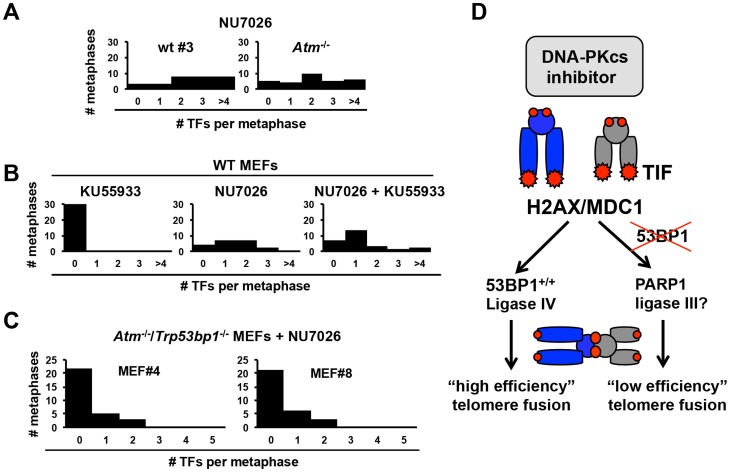
H2AX and MDC1 but not ATM mediate telomere fusions in DNA-PKcs-inhibited cells. (**A**) MEFs deficient for ATM and wt controls were treated with NU7026 for 24 hours and the number of fusions per metaphase quantified by telomere FISH. (**B**) Wild-type MEFs were treated with either NU7026, ATM inhibitor KU55933, or both for 24 hours and telomere fusions were quantified by telomere FISH on metaphase spreads. (**C**) MEFs deficient for both 53BP1 and ATM (*Trp53bp1* ^−/−^/*Atm* ^−/−^) were treated with NU7026 and metaphases analyzed for telomere fusions. (**D**) Schematic of the genetic requirements for DNA-PKcs-inhibited telomeres. H2AX and MDC1 but not ATM normally promote replication-dependent telomere fusions. In cells proficient for 53BP1, fusions are mediated via ligase IV (“high efficiency” C-NHEJ). In the absence of 53BP1, a PARP1-dependent pathway mediates residual fusions (“low efficiency” A-NHEJ).

To determine whether ATM may be required for the fusion of DNA-PKcs-inhibited telomeres, we first quantified telomere fusions in NU7026-treated wt and *Atm* ^−/−^ MEFs (Table 2, Fig. 4A). Interestingly, *Atm* ^−/−^ cells showed similar frequency and distribution of fusions as wt cells examined in parallel (2.2 and 2.7 fusions per metaphase, respectively; Table 2, Fig. 4A). Moreover, co-treatment of wt MEFs with NU7026 and KU55933, a widely employed highly specific ATM catalytic inhibitor, had no measurable effect on the frequency of telomere fusions, relative to treatment with NU7026 alone (1.2 and 1.3 fusions/metaphase, respectively; Table 2, Fig. 4B). As expected [35], control untreated *Atm* ^−/−^ and KU55933-treated wt MEFs did not show telomere fusions (Fig. 4A–B and data not shown).

Because our studies above suggest that telomere fusions in 53BP1-deficient cells occur via a distinct ligase IV-independent, PARP-dependent pathway (Fig. 2), we next examined a specific requirement for ATM in this pathway. To this end, we obtained MEFs double-deficient for 53BP1 and ATM (*Trp53bp1* ^−/−^/*Atm* ^−/−^ MEFs) by timed matings of *Trp53bp1* ^−/−^/*Atm* ^+/−^ mice (previously generated by us [54]). Although *Trp53bp1* ^−/−^/*Atm* ^−/−^ MEFs underwent premature senescence (similar to *Atm^−/−^* MEFs; not shown), we were able to obtain passage-immortalized MEFs from 2 independent embryos for our studies (AB-4 and AB-8, Table 2). Similar to *Atm^−/−^* and *Trp53bp1* ^−/−^ control MEFs, *Trp53bp1* ^−/−^/*Atm* ^−/−^ MEFs did not accumulate telomere fusions (data not shown), suggesting that mouse telomeres remain capped in the absence of both ATM and 53BP1. Significantly, exposure to NU7026 induced fusions with similar frequency and distribution in *Trp53bp1* ^−/−^/*Atm* ^−/−^ MEFs and control *Trp53bp1* ^−/−^ MEFs (23 fusions in 40 *Trp53bp1* ^−/−^/*Atm* ^−/−^ metaphases, or 0.57 fusions per metaphase; data pooled from n = 2 MEF cultures established from 2 independent embryos; Table 2, Fig. 4C). As observed for *Trp53bp1* ^−/−^ MEFs, co-treatment with olaparib abrogated telomere fusions in *Trp53bp1* ^−/−^/*Atm* ^−/−^ MEFs (Table 2), suggesting that residual fusions are dependent on a PARP-regulated pathway. Altogether, these experiments indicate that ATM is dispensable for both the canonical (53BP1-dependent, PARP-independent) and the alternative (53BP1-independent, PARP-dependent) end-joining pathways that fuse telomeres in DNA-PKcs-inhibited cells.

To investigate a requirement for ATM substrates H2AX and MDC1 in the fusion of DNA-PKcs-inhibited telomeres, we quantified telomere fusions in metaphase spreads of NU7026-treated *H2afx* ^−/−^ and *Mdc1* ^−/−^ MEFs. In marked contrast to loss of ATM, loss of MDC1 or H2AX abrogated fusion formation, even though telomere fusions were readily detected in wt MEFs treated and analyzed in parallel (Table 2).

## Discussion

Here, we have taken a genetic approach to investigate the pathways that mediate end-to-end chromosomal rearrangements arising in DNA-PKcs-deficient cells. Our studies demonstrate a key role for 53BP1 in the fusion of DNA-PKcs-deficient telomeres throughout the cell cycle. Moreover, this work revealed a PARP1-dependent pathway that mediates telomere fusions in 53BP1-deficient cells at lower efficiency, consistent with the activation of alternative end-joining. Finally, we demonstrate a requirement for TIF factors H2AX and MDC1 but not ATM in the generation of replication-related fusions at DNA-PKcs-inhibited telomeres.

Previous studies indicated that both the type of DNA lesion and the stage of the cell cycle dictate the choice of end-joining pathway at dysfunctional telomeres. In this context, the fusion of mouse or human telomeres uncapped via TRF2 depletion or inhibition requires both ligase IV [55], [56] and 53BP1 [23]. These observations are in line with others in the context of programmed DSBs, where 53BP1 similarly functions in an epistatic manner with canonical ligase IV-dependent NHEJ to synapse distant DSBs [43], [57]. Here, we show that ligase IV-dependent fusion of DNA-PKcs-deficient telomeres is similarly dependent on 53BP1. Moreover, analysis of cells depleted of both 53BP1 and ligase IV suggest an epistatic relationship in this context as well. Although the specific mechanism by which 53BP1 promotes C-NHEJ at telomeres is not well understood, it has been suggested that 53BP1 functions upstream of Ligase IV to promote chromatin mobility of distant telomeres from nonhomologous chromosomes [23].

Unexpectedly, we found that loss of 53BP1 diminished but did not abrogate telomere fusions in DNA-PKcs inhibitor-treated cells. Moreover, although the pathway mediating fusions in 53BP1-deficient cells was clearly less efficient than the pathway operating in wt cells, it was nevertheless quantifiable using a cytogenetic assay. 53BP1-independent telomere fusion formation has been previously described in contexts other than DNA-PKcs deficiency. For example, ligase IV-independent rejoining of telomeres was previously reported in human telomeres lacking Ku86 (where it mediates sister chromatid fusions) [55], telomeres depleted of POT1 [9], telomeres lacking all shelterin components [18] and critically short telomeres [9]. When analyzed [9], these studies also found that ligase IV-independent telomere fusions do not require 53BP1.

Furthermore, loss of 53BP1 may play an active role in the promotion of A-NHEJ at telomeres when C-NHEJ is absent. In this context, A-NHEJ is thought to require end-resection (presumably to uncover areas of microhomology) and loss of 53BP1 enhances end-resection at DSBs in multiple contexts [18], [58]–[61]. Specifically at telomeres, loss of 53BP1 in shelterin-depleted cells leads to formation of single-stranded DNA and PARP-dependent fusions [18], two hallmarks of A-NHEJ [17]. Together with our findings here, these data suggests that loss of 53BP1 at DNA-PKcs-deficient telomeres may promote their fusion via alternative end-joining via two mechanisms: persistent uncapping due to loss of C-NHEJ and defective end-protection. In the future, it will be interesting to investigate whether this role for 53BP1 is specific to uncapped telomeres or reflects on a more general role in the fusion of broken DNA ends to form chromosomal translocations. In addition, given that the frequency of telomere fusions in DNA-PKcs-inhibited, 53BP1-deficient cells is low, it will be important to develop more robust assays to further dissect the underlying genetic pathway.

Finally, we have examined here roles for several key components of the DNA Damage Response (DDR) in detection and signaling of dysfunctional telomeres prior to their rejoining. We were particularly interested in ATM, as this kinase was previously shown to promote telomere fusions in G1, but to repress (mildly) telomere fusions during replication at TRF2-depleted telomeres [51]. Our analysis of ATM-deficient or ATM-inhibited cells indicates that ATM is also fully dispensable for telomere fusions in DNA-PKcs-inhibited cells. Moreover, analysis of MEFs double deficient for 53BP1 and ATM indicates that ATM is also dispensable for the “low efficiency” PARP-dependent pathway that mediates fusions in 53BP1-depleted cells. In contrast, loss of either H2AX or MDC1, which function to promote NHEJ at “general” and programmed DSBs [62], [63] and at dysfunctional telomeres [53], resulted in a dramatic reduction in telomere fusions. The finding that loss of either H2AX or MDC1 impairs telomere fusion formation to a greater extent than loss of 53BP1 suggests that H2AX and MDC1 function upstream of both the canonical and alternative end-joining pathways.

Lastly, these studies have significant translational implications. Currently, DNA-PKcs inhibitors are undergoing preclinical evaluation for a potential use as tumor radio- and chemosensitizers [64]. Moreover, human cancers with inactivating mutations in DNA-PKcs [65], [66] or 53BP1 [67]–[70] have been described. For example, a recent analysis of the mutational landscape across 12 major cancer types found that mutations in DNA-PKcs are common in bladder, colorectal, lung and endometrial cancers, where they correlate with higher overall mutational load [66]. Moreover, many human tumors favor repair via A-NHEJ even in the absence of known mutations in the canonical pathway [17], [71], [72]. If telomere fusions are a feature of these genetic backgrounds, they may represent a mechanism for tumor evolution and resistance to therapy. In this context, we recently reported that telomere fusions promote T cell lymphomagenesis in DNA-PKcs null mice by generating aneuploidy [8]. Our findings here would predict that alterations in several DNA Damage Response (DDR) factors, such as 53BP1, H2AX and/or MDC1, would have a dramatic effect on the frequency and mechanisms of telomere fusions in tumors with mutant DNA-PKcs or treated with a DNA-PKcs inhibitor.

## Supporting Information

Figure S1 Examples of NU7026-induced telomere fusions in metaphase spreads of Trp53bp1+/+ and Trp53bp1−/− MEFs. See Figure 1B for magnifications.
(PDF)
