# Supplementary material for: 53BP1 Mediates the Fusion of Mammalian Telomeres Rendered Dysfunctional by DNA-PKcs Loss or Inhibition
Source: PLoS One. 2014 Sep 29;9(9):e108731. doi: 10.1371/journal.pone.0108731 (PMC4181871; doi:10.1371/journal.pone.0108731)

Figure S1. Examples of NU7026-induced telomere fusions in metaphase spreads of *Trp53bp1*$^{+/+}$ and *Trp53bp1*$^{-/-}$ MEFs. See Figure 1B for magnifications.

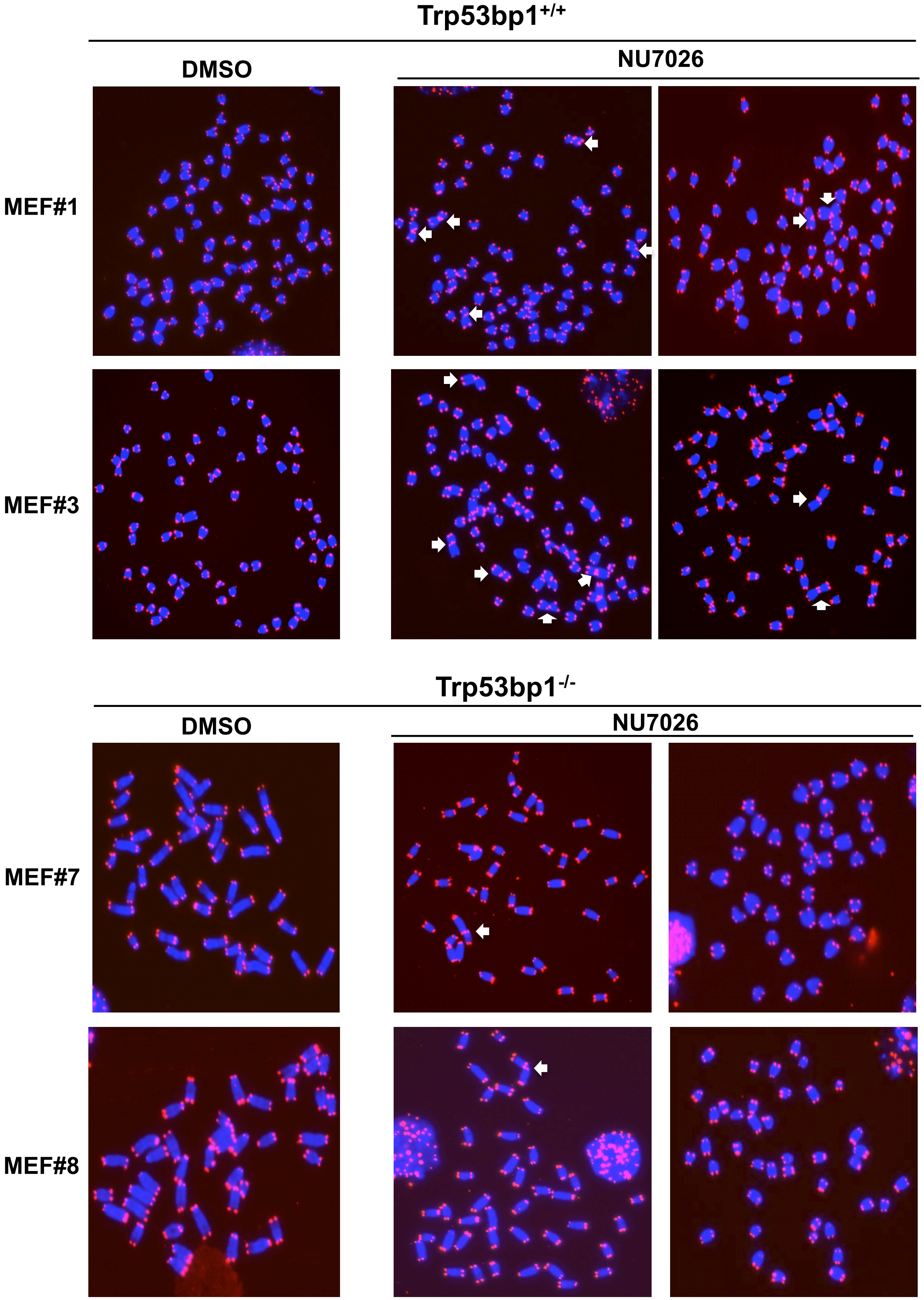

Supplement: Figure S1 — Examples of NU7026-induced telomere fusions in metaphase spreads of Trp53bp1+/+ and Trp53bp1−/− MEFs. See Figure 1B for magnifications. (PDF) [file pone.0108731.s001.pdf]
